# βeta-2 glycoprotein I is a novel regulator of Apolipoprotein E containing HDL particles in females

**DOI:** 10.1186/s13293-025-00766-9

**Published:** 2025-10-17

**Authors:** Ying Wang, Miao Qi, Liming Chen, Maaike Kockx, James Weaver, Leonard Kritharides, Steve Krilis, Bill Giannakopoulos

**Affiliations:** 1 Department of Infectious Disease, Immunology and Sexual Health, St George Hospital, Kogarah, Sydney Australia; 2 Faculty of Medicine and Health, School of Clinical Medicine, University of New South Wales, St George Hospital Campus, Kogarah, Sydney, Australia; 3 NHC Key Lab of Hormones and Development and Tianjin Key Lab of Metabolic Diseases, Tianjin Medical University Chu Hsien-I Memorial Hospital & Institute of Endocrinology, Tianjin, China; 4 Anzac Research Institute, Concord Repatriation General Hospital and University of Sydney, Concord, Sydney, NSW Australia; 5 Department of Cardiology, St George Hospital Kogarah, Sydney, Australia; 6 Department of Cardiology, Concord Hospital, Sydney, Australia; 7 Department of Rheumatology, St George Hospital, Kogarah, NSW Australia

**Keywords:** Apolipoprotein H, Β2-glycoprotein I, Apolipoprotein E, High density lipoprotein, Β2-glycoprotein I deficiency

## Abstract

**Background:**

βeta-2 glycoprotein I (β2GPI, Apolipoprotein H) is a plasma glycoprotein best known as a major autoantigen in autoimmune disorders such as antiphospholipid syndrome (APS) and systemic lupus erythematosus (SLE), both of which confer elevated cardiovascular risk. Despite its prominence in autoimmunity, its role in lipid metabolism and potential sex-specific effects remain poorly understood.

**Methods:**

We investigated β2GPI’s influence on lipoprotein profiles using β2GPI knockout (KO) and wild-type (WT) mice subjected to normal chow (NC) and high-fat (HF) diets, as well as plasma from β2GPI-deficient patients and aged and sex matched controls. Lipoprotein fractions were analyzed for cholesterol and apolipoprotein content, and protein interactions were assessed by co-immunoprecipitation.

**Results:**

In animal studies, female β2GPI KO mice—but not males—displayed significantly increased total plasma cholesterol on a HF diet and greater cholesterol content within HDL fractions. Apo E was enriched in HDL fractions from female KO mice under both NC and HF diets, and plasma Apo E was elevated in HF-fed female KOs. In WT females on HF diet, β2GPI was enriched in HDL fractions, and β2GPI co-immunoprecipitated with Apo E. In human studies, the β2GPI-deficient female patient exhibited increased HDL cholesterol, a shift toward larger HDL particles, and enriched Apo E in HDL fractions relative to controls. Co-immunoprecipitation confirmed β2GPI–Apo E interaction in human plasma, with binding requiring Domain V of β2GPI.

**Conclusions:**

Our findings identify β2GPI as a sex-specific regulator of HDL metabolism. In females, β2GPI modulates Apo E-containing HDL particles, influencing cholesterol distribution and lipoprotein composition. These results reveal a novel mechanism linking β2GPI to lipid homeostasis, with potential implications for cardiovascular risk in women with autoimmune disease. Targeting β2GPI–Apo E interactions may represent a therapeutic avenue for correcting dysregulated HDL metabolism in female-specific cardiometabolic and autoimmune contexts.

## Highlights

- β2GPI (Apolipoprotein H) regulates HDL cholesterol levels in a sex-specific manner, significantly impacting lipid metabolism in females but not males.
- Female β2GPI knockout mice exhibit elevated total cholesterol and enrichment of ApoE in HDL particles, especially under high-fat diet conditions.
- β2GPI directly interacts with ApoE, with domain V of β2GPI mediating this binding in both mouse and human plasma.
- A rare β2GPI-deficient female patient showed larger HDL particle size, mirroring the murine phenotype.
- Findings suggest β2GPI is a novel modulator of ApoE-containing HDL particles in females, with potential relevance to cardiovascular and autoimmune diseases, and possibly Alzheimer’s disease risk.

## Layperson Summary

This study uncovers a new role for a blood protein called β2-glycoprotein I (also known as Apolipoprotein H) in cholesterol metabolism, specifically in females. β2-glycoprotein I is best known for its involvement in autoimmune diseases such as lupus and antiphospholipid syndrome — conditions that mostly affect women and increase cardiovascular risk. Using both mouse models and rare human samples, we found that the absence of β2-glycoprotein I leads to increased levels of certain high-density lipoprotein (HDL) particles in females, but not in males. These HDL particles also showed higher levels of another key protein called Apolipoprotein E (ApoE), which is involved in cholesterol transport and linked to diseases like Alzheimer’s. However, not all HDL particles have the same biological effects — some may be protective, while others could contribute to disease. Our findings suggest that β2-glycoprotein I may play an important role in maintaining the balance of HDL particle types in females. Disrupting this balance could affect how cholesterol is processed in the body, with implications for heart and brain health. This research helps explain important sex differences in disease risk and opens new paths for targeted treatment strategies, particularly for women.

## Background

The metabolism and function of lipoproteins and triglycerides are crucial for cardiovascular health [1]. Lipoproteins transport lipids in the bloodstream and triglycerides are a primary form of stored fat and a source of energy. Dysregulation of these molecules is linked to metabolic disorders like atherosclerosis, diabetes, and obesity.

Apolipoprotein H (Apo H), also known as β2-glycoprotein I (β2GPI), is an abundant plasma protein involved in coagulation and immune responses [2]. β2GPI binds negatively charged molecules such as anionic phospholipids [3]. Its role in triglyceride metabolism remains controversial, which may be due to differences in the ethnic background of the different populations studied [4, 5]. Studies that have looked at the HDL proteome in humans and rats have noted that β2GPI is one of the relatively abundant proteins identified specifically in small HDL particles which have distinct protein signatures and biological functions [6, 7]. The HDL Proteome Watch website (http://homepages.uc.edu/~davidswm/HDLproteome.html*)* confirms β2GPI as a HDL apolipoprotein [8, 9]. The distinct biological functions attributed to HDL particles, including anti-oxidant, hemostasis, and anti-inflammatory have also been attributed to β2GPI when it has been studied in isolation, including heparin binding, [10] anti-oxidant, [11, 12] anti-inflammatory in the context of lipopolysaccharide induced inflammation, [13] and anti-thrombotic properties [14]. Rodent studies suggest β2GPI may influence lipid metabolism by modulating lipoprotein lipase activity, [15] though others have not reproduced these results [16]. 

Our study aims to definitively elucidate the role of β2GPI on lipoprotein and triglyceride levels utilising male and female mice with and without the β2GPI gene fed either normal chow or high fat diet. We also aim to examine the validity of our studies to human physiology by comparing plasma samples from normal and β2GPI completely deficient individuals who we have previously characterised [17]. 

In summary, understanding β2GPI’s role in lipid metabolism is essential for addressing related pathologies.

## Methods

### Animals

Eight weeks old C57BL/6 Wild Type (WT) and β2GPI knockout (KO) mice on a C57BL/6 background were used in this study. All the animals were housed in an environment with a temperature of 20 ± 2 °C, relative humidity of 50 ± 1%, and a light/dark cycle of 12/12 hr. All mice had ad libitum access to water and food. All animal studies were done in compliance with the regulations and guidelines of St George Hospital, University of New South Wales institutional animal care and conducted according to the AAALAC and the IACUC guidelines (application number: 4269752/2 and approval number: 14/84B).

Mice were divided into 8 groups (4 or 5 mice per group, according to sex and genotype): (1) WT female mice fed a normal chow (NC) diet; (2) β2GPI KO female mice fed a NC diet; (3) WT male mice fed a NC diet; (4) β2GPI KO male mice fed a NC diet; (5) WT female mice fed a high fat (HF) diet; (6) β2GPI KO female mice fed a HF diet; (7) WT male mice fed a HF diet; (8) β2GPI KO male mice fed a HF diet. Each group was treated with a HF or NC diet for 16 weeks. The NC diet was obtained from Gordon’s Specialty Stockfeeds, NSW, Australia, and contained 11 kJ/g; energy 14% fat, 21% protein and 65% carbohydrate, while the HF diet was SF00-219, from Specialty Feeds, Glen Forest, Western Australia, and contained 19.4 kJ/g; energy 40% fat, 17% protein, 43% carbohydrate. The mice were fasted overnight for 10–14 h before euthanasia and blood collection. They were weighed every week, and food intake was measured weekly by monitoring the weight of the remaining food as performed previously [18].

### Sample collection

#### Mice

Mouse blood samples were collected by cardiac puncture in 1-mL tubes containing K3 EDTA. (Greiner Vacuette Minicollect K3 EDTA tube; Greiner Bio-one GmbH, Kremsmünster, Austria). Blood samples were centrifuged (1,000 g for 10 min), and the plasma fractions were collected and stored at − 80 °C.

#### Human samples

We identified 3 individuals that did not have detectable β2GPI using a highly sensitive β2GPI ELISA in our recent study of 596 patients with coronary artery disease from St George Hospital campus, University of New South Wales. The study was approved by the local Human Research Ethics Committee and was listed in the Australian New Zealand Clinical Trials Registry (ACTRN12616001312437) [17]. Inclusion and exclusion criteria are detailed in the study [17]. Of the three β2GPI deficient plasma samples used in the current study, one was from a female, and two were from males. Plasma samples from age and sex matched controls, 3 females and 4 males from the same study were also obtained [17]. Samples were collected by venipuncture into 5 ml tubes containing EDTA (BD Vacutainer blood collection tubes, BD, Becton Dickinson Franklin Lake, NT). They were centrifuged (1,000 g for 10 min), and the plasma fractions were collected and stored at − 80 °C.

### ELISAs for plasma Apo E and β2GPI levels

Plasma levels of Apo E were assessed using specific ELISA kits (Lifespan Biosciences, Seattle, WA) according to the manufacturer’s instructions. Total β2GPI mouse and human plasma levels were analyzed as previously described [13, 19].

### Quantification of mouse plasma cholesterol and triglyceride levels

Total cholesterol and triglycerides were enzymatically quantified using commercial kits (Wako Chemicals, Richmond, VA).

### Fast protein liquid chromatography (FPLC)

Lipoprotein fractions in mouse and human plasma were separated by FPLC using a Superose 6 10/300 GL column (GE Healthcare). Plasma samples were loaded onto the column and eluted with 20 mM sodium phosphate buffer/0.5 M NaCl, pH 7.8 at a flow rate of 0.25 mL/min. Absorbance was monitored at 280 nm, after which 250 µl fractions were collected over 1 h as previously described [20].

### Western blot analysis for Apo E, Apo AI, and β2GPI levels in plasma and lipoprotein fractions

Briefly, equal protein amounts of plasma or lipoprotein fractions from individual mice were subjected to SDS-PAGE and Western blot analysis using primary antibodies to mouse Apo E (polyclonal rabbit anti-mouse, 1:2000 dilution), mouse Apo AI (polyclonal rabbit anti-mouse,1:2000 dilution) or an anti-β2GPI rabbit polyclonal antibody (1:2000 dilution) generated in house [21]. The anti-Apo E and anti-Apo AI antibodies were obtained from Abcam (Cambridge, MA). An identical plasma and lipoprotein fractions analysis was performed in 3 individuals with β2GPI deficiency and 7 (3 females and 4 males) age and sex-matched controls. The secondary antibodies (goat anti-rabbit and rabbit anti-goat HRP-labeled, 1:2000 dilution) were obtained from Dako, (Santa Clara, CA). Blots were developed with enhanced chemiluminescence (ECL) reagent (Amersham ECL™ Western Blotting Reagents, Buckinghamshire, UK), analyzed as above. WT mouse plasma, 1:200 dilution, served as the control for the mouse studies.

### Co-immunoprecipitation experiments with WT and β2GPI-deficient mouse and human plasma

Plasma samples (100 µl) were diluted 1:5 with 400 µl of PBS. Samples were pre-cleared by incubating with 115 µl of protein G-Sepharose beads (Merck Millipore) for 2 h at 4℃ to remove nonspecific binding. Pre-cleared plasma was obtained following centrifugation for 30 s at 14,000 g. Individual plasma samples were then incubated for 14–16 h at 4℃ with either sheep anti-mouse β2GPI antibody (R&D systems) (10 µg/ml) or rabbit anti-human β2GPI antibody, [22] at 5 µg/ml final antibody concentration, [23] followed by the addition of 20 µl of protein G-Sepharose beads. The samples were then centrifugated for 5 s at 14,000 g at 4℃ in an Eppendorf microfuge and washed six times with 500 µl PBS. After the final wash, the protein G-containing immunoprecipitates were resuspended in SDS-PAGE sample buffer with β-mercaptoethanol, boiled for 5 min at 100℃ and then separated on a 4–12% Bis-Tris gel (Invitrogen). A total of 2 µl plasma from either 1 patient with β2GPI deficiency and 1 control or pooled samples from 5 female C57BL/6 WT and 5 β2GPI KO mice were subjected to SDS-PAGE and served as positive and negative controls, respectively. The proteins were transferred to PVDF membranes and immunoblotted with rabbit anti-mouse (1:2000) or anti-human ApoE (1:2000), or rabbit anti-mouse (1:2000), or anti-human β2GPI antibodies (1:2000).

### Binding of Apo E to human β2GPI and domain deletion mutants DI-DIV and DII-DV

Human Apo E2 (1 mg/ml) (Abcam, Cambridge, MA) was labeled by EZ-Link™ Sulfo-NHS-Biotinylation Kit (Thermo Scientific, Waltham, MA) according to the manufacturer’s instructions. The efficiency of biotin labeling was checked by Western blot (data not shown). Human β2GPI and domain deletion mutants DI-DIV and DII-V (expressed in a baculovirus system in our laboratory as previously described [14] were coated on Maxisorb plates (Thermo Scientific) overnight at 4 °C at a concentration of 1 µM. Plates were washed 4 times with PBS–0.1% Tween 20 and then blocked with 2% BSA/PBS–0.1% Tween 20 for 1 h at RT [21]. After washing, Apo E-Biotin (50 nM) was added to each well and incubated for 1 h at RT. After washing 4 times with PBS–0.1% Tween 20, streptavidin-HRP (Dako) was added (1:2000 dilution) and incubated for 1 h at RT. After washing x4 the plates were read at an optical density (OD) 450 after the addition of the chromogenic substrate 3,3’,5,5’- tetramethylbenzidine (TMB) (Sigma).

### Statistical analysis

GraphPad Prism version 8 was used for statistical analysis and data plotting. All data were presented as mean ± standard deviation. For data with normal distribution, an unpaired Student t-test was used for group comparisons. For non-parametric data, the Mann-Whitney test was used. A p-value < 0.05 represents statistical significance.

Statistical analysis was performed separately for males and females. This sex-stratified approach was prespecified because the primary aim of the study was to determine whether β2GPI deficiency exerts female-specific effects on lipoprotein metabolism. Given our sample size of 5 mice per sex/genotype/diet group, this strategy provided the most direct and interpretable comparisons (WT vs. KO within each sex and dietary condition) while avoiding unstable interaction estimates from an underpowered factorial model, Such as 3 way ANOVA.

## Results

### Animal studies

#### Total plasma cholesterol increases in female C57BL/6 β2GPI deficient (KO) mice fed a HF diet

**Fig. 1:**
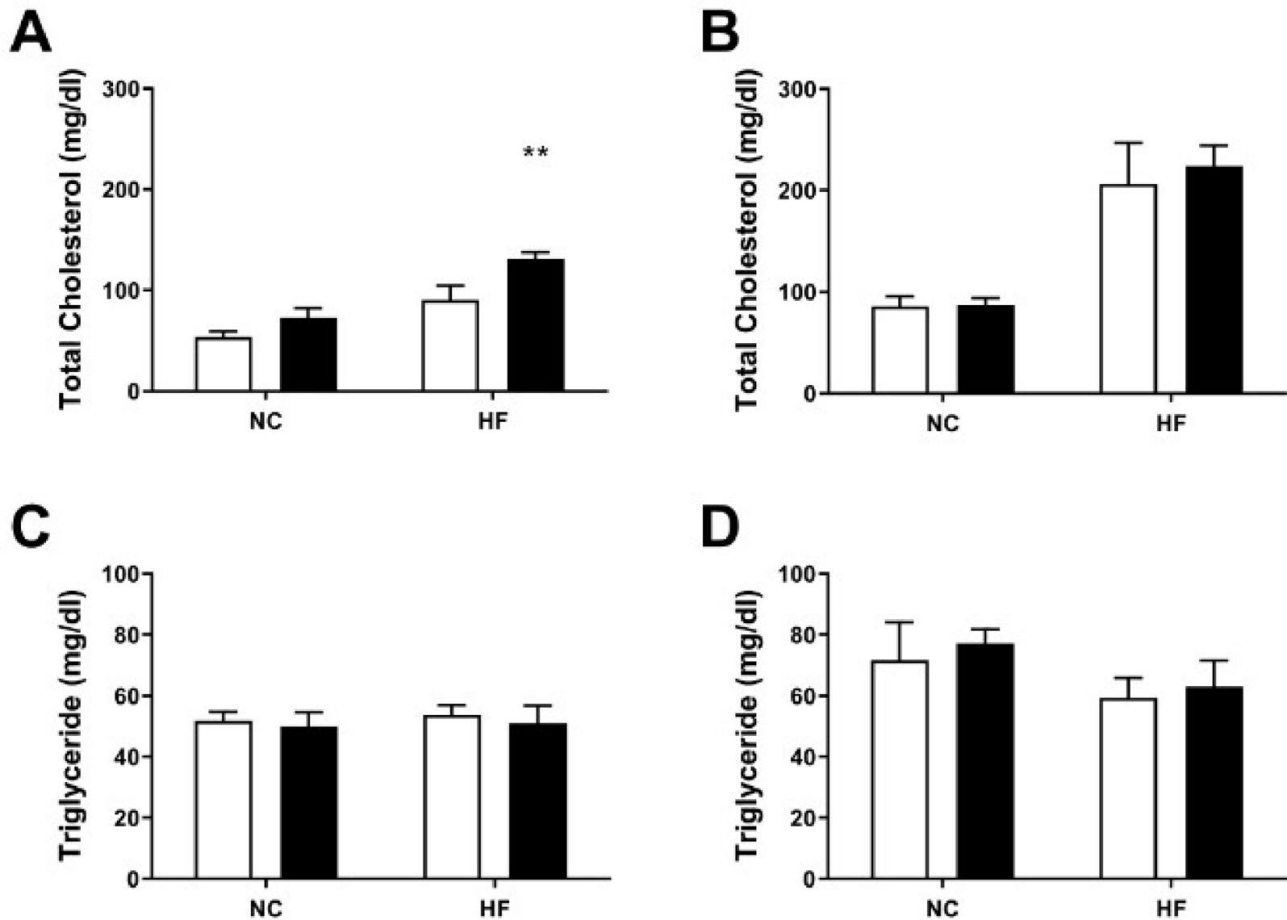
The total cholesterol (mg/dl) of (**A**) female (**B**) male WT and β2GPI KO mice fed with NC or HF diet, respectively. The triglyceride concentration (mg/dl) of female (**C**) and male (**D**) WT and β2GPI KO mice when fed with NC or HF diet, respectively. *n* = 5, *p < 0.05 by two tailed Student’s t-test. WT = wild type, mg/dl = milligrams/decilitre  = WT,  = β2GPI KO, NC= Normal Chow, HF= high fat, KO = Knockout

WT and β2GPI KO female and male mice were maintained on a NC or HF diet for 16 weeks. Compared to female WT mice, female β2GPI KO mice fed a HF diet had a higher total cholesterol (Figs. 1A 136.73 ± 4.04 mg/dl vs. 90.05 ± 14.41 mg/dl, *n* = 5, *p* < 0.05). There was no difference in total cholesterol between female β2GPI KO mice and female WT mice fed a NC diet (Fig. 1A 72.96 ± 9.42 mg/dl vs. 53.65 ± 5.76 mg/dl, *n* = 5, *p* > 0.05). Also, there was no difference between male β2GPI KO and male WT mice fed a HF diet (Fig. 1B 223.98 ± 20.48 mg/dl vs. 206.34 ± 40.43 mg/dl, *n* = 5, *p* > 0.05) or NC diet (Fig. 1B and 87.35 ± 6.58 mg/dl vs. 85.88 ± 9.76 mg/dl, *n* = 5, *p* > 0.05). Furthermore, no difference was found in triglyceride concentration in female or male β2GPI KO mice compared to female and male WT when fed a NC or HF diet (Fig. 1C, D).

### Female β2GPI KO, but not male β2GPI KO mice have increased cholesterol content in the HDL fractions

**Fig. 2:**
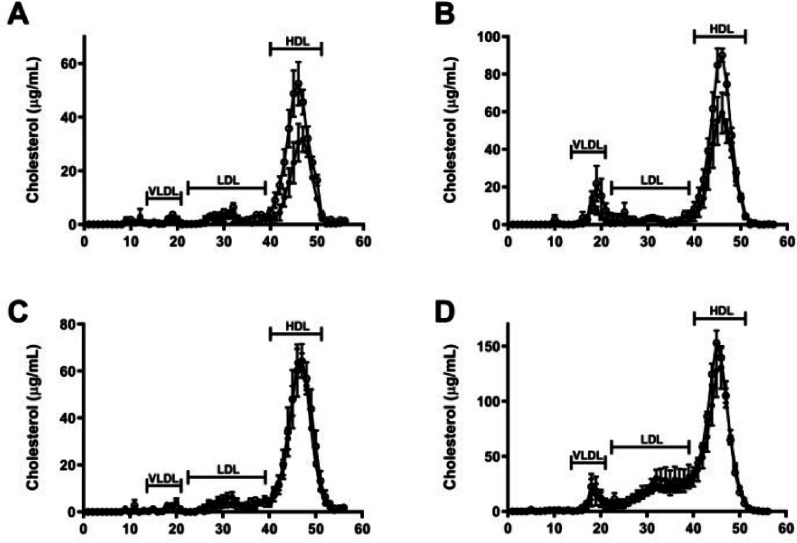
Lipoprotein separation of plasma samples from WT and β2GPI KO female and male mice fed a HF or NC diet using FPLC. (**A-D**) (**A**) Female WT and β2GPI KO mice fed a NC and (**B**) HF diet (*n* = 5). (**C**) Male WT and KO mice fed NC (*n* = 5) and (**D**) HF diet (*n* = 4). Cholesterol levels are expressed in µg/ml. The numbers on the X axis denote FPLC fractions. VLDL eluted in fractions 13 to 21, LDL in fractions 22 to 39 and HDL in fractions 40 to 51. WT = wild type, KO = Knockout, NC = normal chow, HF= high fat  = β2GPI KO  = WT

FPLC was used to separate the lipoproteins in plasma (Fig. 2). Previous studies have demonstrated that the different lipoprotein classes elute in different fractions [24]. In our study 56 fractions were collected, corresponding to the different lipoproteins. 13–21 (VLDL), 22–39 (LDL) and 40–51 (HDL).

As is usual in mice, HDL was the main carrier of cholesterol in plasma. There was a clear difference in the HDL levels between female WT and β2GPI KO mice on NC and HF diet (Fig. 2A, B), while there was no difference in the HDL levels in the male WT compared to β2GPI KO mice fed a NC or HF diet (Fig. 2C, D). Furthermore, the HDL levels were increased in female β2GPI KO mice fed a HF diet (Fig. 2B) and eluted earlier in the female β2GPI KO mice fed a NC diet (Fig. 2A), suggesting a shift to larger HDL particles.

### Apo E levels were increased in the HDL fractions in the female β2GPI KO mice fed a NC or HF diet

**Fig. 3:**
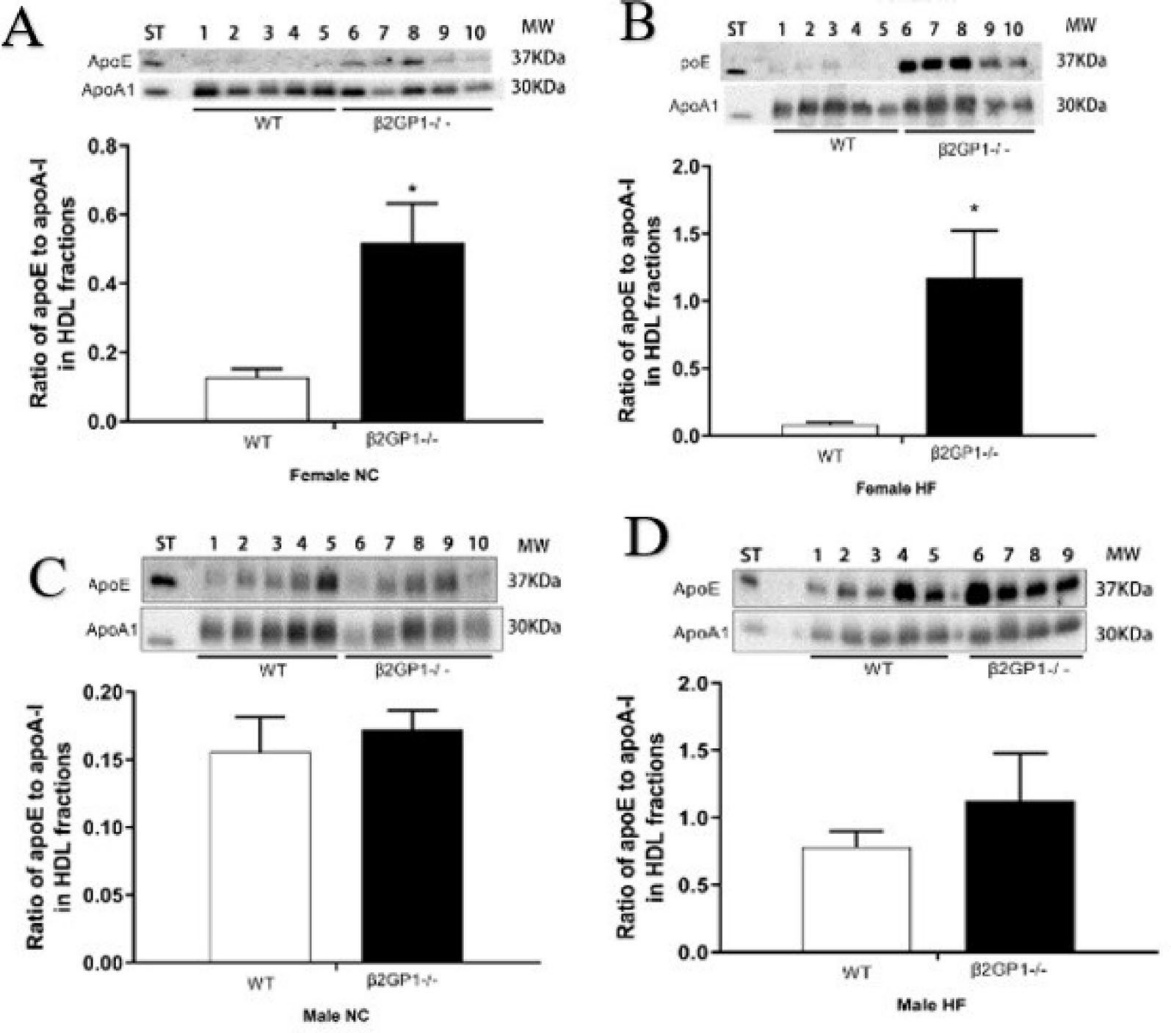
Western blot analysis of ApoE and ApoAI from individual HDL FPLC fractions comparing WT and β2GPI KO mice fed NC or HF diet. (**A**) Immunoreactivity of ApoE and ApoAI in FPLC HDL fractions from individual mice, 5 WT and 5 β2GPI KO (**A**) female mice fed NC (**B**) female mice fed a HF diet. (**C**) male mice fed NC. (**D**) male mice fed a HF diet. The ratio of Apo E to Apo AI in the HDL fractions are shown in the bar graphs below the Western blots (**A**), (**B**), (**C**), and (**D**) respectively. WT = Wild type, KO = β2GPI Knockout, NC = normal chow, HF = high fat,  = WT,  = β2GPI KO, ST = standard (normal plasma), Apo E = Apolipoprotein E, Apo AI = Apolipoprotein AI, MW = molecular weight, kDa = kilodaltons

In order to further elucidate the effect of β2GPI on lipids in HDL, two major apolipoproteins, in HDL, Apo E and Apo AI were quantified. FPLC fractions were subjected to Western Blotting (Fig. 3). Apo E was significantly increased in the HDL fractions in female β2GPI KO fed either NC or HF diet compared to female WT mice (Fig. 3A, B). However, Apo E levels were not different in the HDL fractions of both WT and β2GPI KO male mice fed a NC or HF diets (Figs. 3C, D).

Densitometry was performed on the immunoreactive bands on Western blot using individual mouse HDL fractions for Apo E and is expressed as a ratio of the Apo AI the main protein in HDL (Fig. 3A, B, C, D). There was increased Apo E/Apo AI ratio in the HDL fractions of female β2GPI KO fed NC (0.52 ± 0.25, *n* = 5) compared to female WT mice (0.13 ± 0.05, *n* = 5, *p* < 0.05) indicating enrichment of Apo E in HDL in the β2GPI KO mice (Fig. 3A). HF fed β2GPI KO female mice also had a significantly higher ratio (1.17 ± 0.79, *n* = 5) compared to female WT mice (0.08 ± 0.04, *n* = 5, *p* < 0.05) (Fig. 3B). There was no difference in the Apo E/Apo AI ratio in the HDL fractions in the male β2GPI KO compared to male WT mice fed either a NC or HF diet (Fig. 3C, D).

### Apo E levels are increased in the plasma of female β2GPI KO mice fed a HF diet

**Fig. 4:**
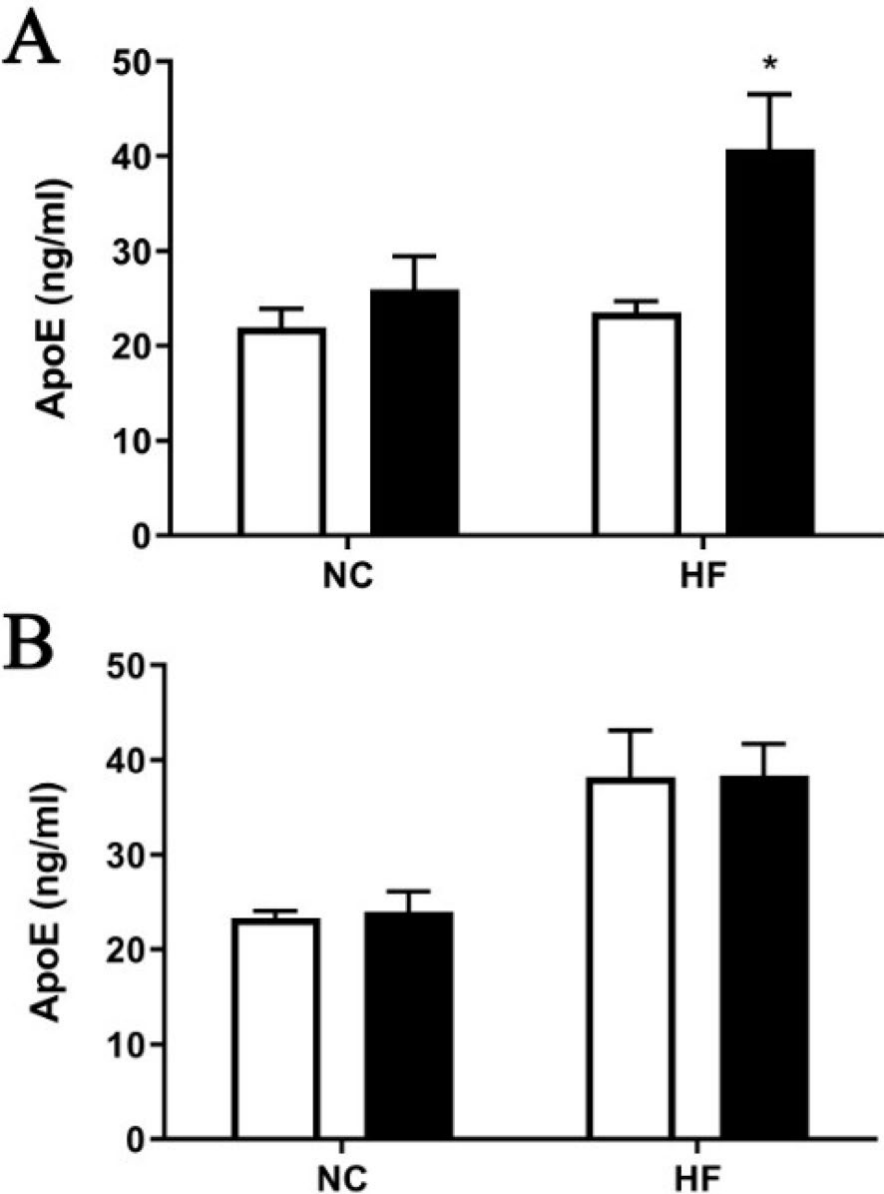
The plasma ApoE concentration of (A) female and (B) male WT and β2GPI KO mice when fed either a NC or HF diet. *n* = 5, *p < 0.05 by two-tailed Student’s t-test. NC = normal chow, HF = high fat diet, Apo E = Apolipoprotein E, ng/ml = nanograms per milliliter **=** WT,  = β2GPI KO

The plasma concentration of Apo E was significantly increased in female β2GPI KO (40.7 ± 5.84 ng/ml, *n* = 5) compared to female WT mice (23.50 ± 2.63 ng/ml, *n* = 5, *p* < 0.05) fed a HF diet but not in females fed a NC diet (Fig. 4A). However, there was no difference in plasmam Apo E levels in male β2GPI KO mice compared to male WT mice fed either NC or HF diet (Fig. 4B).

### β2GPI is enriched in HDL fractions of female WT mice fed a HF diet

**Fig. 5:**
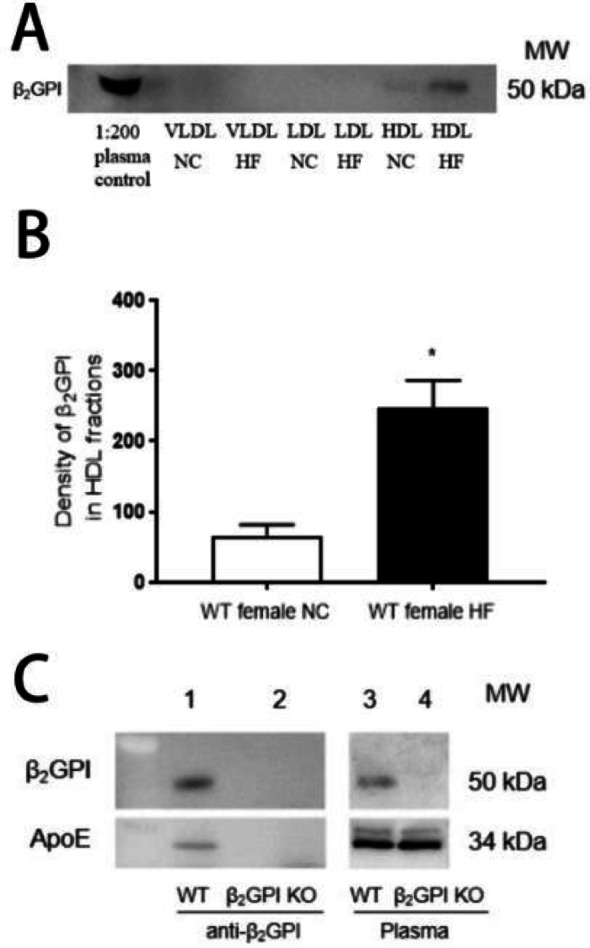
(**A**) β2GPI immunoreactivity in the pooled plasma lipoprotein fractions (VLDL, LDL, HDL) in female WT mice fed NC or HF diet. β2GPI reactive bands were only detected in the HDL fractions. (**B**) Quantifications of the scanned β2GPI immunoreactive bands in the HDL fractions of WT mice. Density of β2GPI in the HDL FPLC fractions from female WT mice fed a NC or HF diet. *n* = 5, *p < 0.05. (**C**) Immunoprecipitation of ApoE with an anti-β2GPI monoclonal antibody using plasma from female WT and β2GPI KO mice. Immunoprecipitates from WT or β2GPI KO mouse plasma was subjected to Western blot using an antibody to ApoE or β2GPI. Immunoreactive bands for β2GPI and ApoE were detected in the immunoprecipitates from WT (lane 1) but not from β2GPI KO mice (lane 2). Plasma controls consisted of WT mouse plasma demonstrating immunoreactive bands to β2GPI and ApoE (lane 3), β2GPI KO plasma did not have an immunoreactive band to β2GPI but had an immunoreactive band to ApoE (lane 4). VLDL = Very low density lipoproteins, LDL = low density lipoproteins, HDL = High density lipoproteins, KO = Knockout, HF = high fat diet, NC = normal chow, WT = Wild type

As our findings pointed to an enrichment of Apo E in HDL in female β2GPI KO mice, we next investigated the distribution of β2GPI amongst the lipoprotein fractions in plasma from WT mice. FPLC fractions from pooled peaks corresponding to VLDL, LDL, and HDL from female WT mice fed either NC or HF diet (Fig. 5A) were subjected to Western blotting with an antibody to β2GPI. The anti-β2GPI immunoreactivity was identified in the HDL fractions in the NC and HF diet fed female mice, with a significant increase in the immunoreactivity of β2GPI in the individual peak fractions from the HDL FPLC peak in the female mice fed a HF diet (Fig. 5B).

### β2GPI co-immunoprecipitates with plasma Apo E

For immunoprecipitation experiments using mouse plasma from female WT and KO mice specific immunoreactive bands to Apo E were obtained with WT but not β2GPI KO mice (Fig. 5C, lanes 1, 2.) The plasma from female WT and KO mice had immunoreactive bands for Apo E (Fig. 5C, lanes 3,4). There was no immunoreactivity for either Apo E or β2GPI in the immunoprecipitant of the β2GPI deficient mice (Fig. 5C, lane 2), indicating a direct interaction between Apo E and β2GPI in the plasma of the WT mice.

## Human studies

### The female β2GPI deficient patient demonstrated increase in HDL cholesterol levels and a shift to larger HDL particles compared to the normal controls

**Fig. 6:**
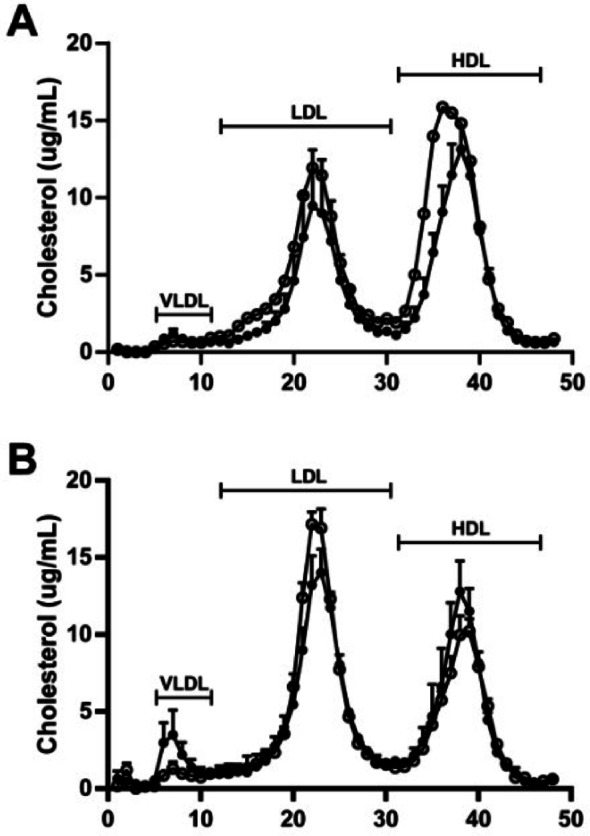
Lipoprotein separation of human plasma samples using FPLC. (**A**) females (**B**) males. Plasma used was from 3 patients deficient in β2GPI (1 female and 2 males) and 7 age and sex matched controls (3 females and 4 males), Cholesterol levels are expressed in µg/ml. The numbers on the horizontal axis denote FPLC fractions. VLDL eluted in fractions 5 to 11, LDL in fractions 12 to 30 and HDL in fractions 31 to 46. VLDL = Very Low-density lipoprotein, LDL = low density lipoprotein, HDL = high density lipoprotein  controls,  β2GPI deficient patients, β2GPI = βeta-2-glycoprotein-I

Human plasma lipoprotein fractions were separated by FPLC (Fig. 6). The major eluting peak was HDL in plasma samples from female patients (Fig. 6A), while LDL had the highest peak in male plasma samples (Fig. 6B). As observed in mice, β2GPI deficiency in the female patient showed higher HDL cholesterol levels and a shift to larger particles, whilst this was not observed for the two male patients examined. (Fig. 6A, B).

### Enriched Apo E in the HDL fractions in the female patient with β2GPI deficiency

**Fig. 7:**
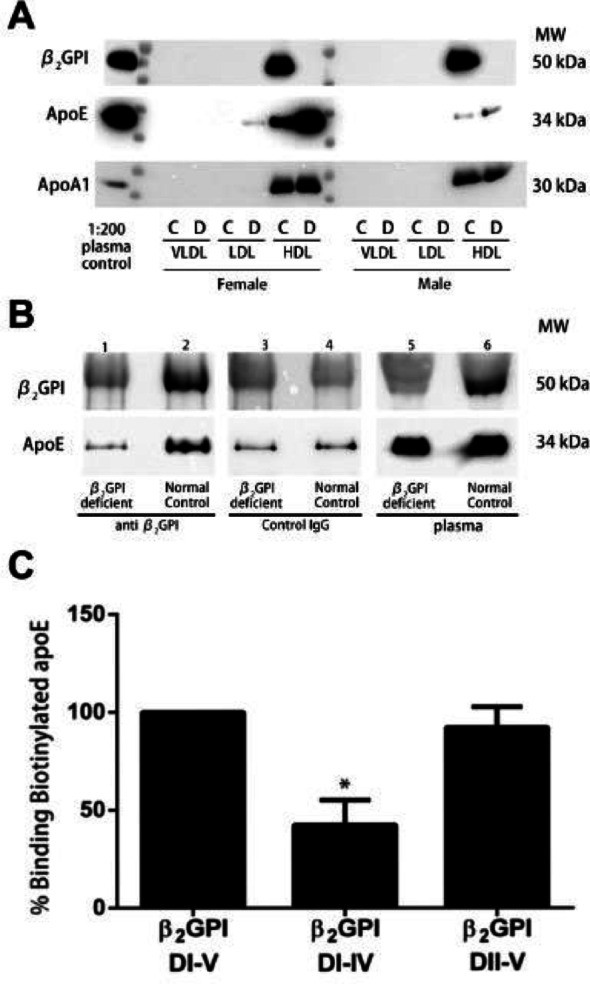
Effect of β2GPI deficiency on levels of ApoE in human HDL plasma samples. (**A)** Western blot analysis of β2GPI, Apo E and Apo AI in pooled VLDL, LDL and HDL FPLC fractions from β2GPI deficient or control patient plasma samples. C = normal controls D = β2GPI deficient (**B**) Immunoprecipitates from one β2GPI deficient patient sample (lanes 1,3) and one normal control (lanes 2, 4) were subjected to Western blot using anti-β2GPI monoclonal antibody (lanes 1,2), or with an isotype control IgG monoclonal antibody (lanes 3, 4) and plasma from a female β2GPI deficient patient (lane 5) or normal control (lane 6). Immunoreactivity to β2GPI was not detected in any of the β2GPI deficient patient samples. In the β2GPI deficient samples ApoE immunoreactivity was only detected in the plasma sample (lane 5) but not in the immunoprecipitation samples (lane 1). Immunoreactivity for β2GPI and ApoE was detected using specific anti-human β2GPI or ApoE antibodies. (**C**) Binding of biotinylated ApoE to β2GPI and domain deletion mutants DI-IV and DII-V. % binding is expressed as a percentage of the binding of ApoE to full length β2GPI (DI-DV) which is set as 100. β2GPI = β2 glycoprotein I, ApoE = Apolipoprotein E, ApoAI = Apolipoprotein A I, MW = molecular weight, kDa = Kilodaltons

Equal protein amounts of plasma or lipoprotein fractions from individual patient samples were separated by FPLC. Patient samples from the VLDL, LDL, and HDL fractions were pooled and subjected to Western blotting (Fig. 7A). The normal control samples immunoreactivity for β2GPI, ApoE and ApoAI were only detected in the HDL fractions (Fig. 7A, C = control, D = β2GPI deficient) indicating enrichment of ApoE in HDL in the β2GPI deficient patient in line with our mouse studies. The Apo E immunoreactive bands in the HDL fractions were increased in the females compared to the male patients (Fig. 7A). The single β2GPI deficient female plasma sample had increased density of the Apo E bands relative to the density of Apo AI bands compared to the pooled female control samples (Fig. 7A). The Apo E immunoreactivity in the β2GPI deficient female plasma sample was also substantially increased compared to the male β2GPI deficient and control plasma samples (Fig. 7A). The ApoAI immunoreactivity was equal in both the female and male controls and β2GPI deficient HDL fractions (Fig. 7A).

### β2GPI co-immunoprecipitated with Apo E in human plasma

Distinct immunoreactive bands were not detected in the Western blot with antibodies to Apo E or β2GPI in the immunoprecipitant samples from the female β2GPI deficient patient (Fig. 7B, lane 1) compared to normal controls (Fig. 7B, lane 2), indicating a direct interaction between Apo E and β2GPI in the plasma. Apo E immunoreactivity was detected in the plasma of β2GPI deficient (Fig. 7B, lane 5) and control samples (Fig. 7B, lane 6). There was no distinct immunoreactivity for β2GPI in the immunoprecipitant of the β2GPI deficient individual and normal controls when control rabbit IgG was used in the immunoprecipitation experiments (Fig. 7B, lanes 3,4). The very faint bands observed in the western blots migrating at the level of β2GPI and ApoE were non-specific (Fig. 7B, lane 1, 3, 4).

### Domain V of β2GPI is required for binding to Apo E

β2GPI mutants were used to identify the specific domain(s) of β2GPI responsible for binding to human Apo E. β2GPI mutants lacking Domain V (DI-IV) demonstrated a significant decrease, compared to full length β2GPI (DI-V), in % binding to Apo E (42.5 ± 12.60 vs. 92.20 ± 10.40, *n* = 5, *p* < 0.05) (Fig. 7C). The mutant lacking DI (DII-V) demonstrated binding equivalent to full length β2GPI (DI-V) (Fig. 7C), indicating that interaction between Apo E and β2GPI occurs through Domain V.

## Discussion

In this study we elucidated a sex-specific role of β2GPI in HDL cholesterol metabolism. The absence of β2GPI in C57BL/6 female mice led to significantly higher levels of total cholesterol, while triglyceride levels remained unchanged. No such differences were observed in male mice between the WT and KO groups. Chromatographic analysis revealed a significant difference in the HDL cholesterol fraction between female WT and β2GPI KO mice, with the KO mice showing higher levels. This difference was observed in both NC and HF diet groups. In contrast, no such separation was seen in the male mice on either diet. Previous studies have shown that the majority of plasma cholesterol in mice is associated with HDL [25]. This suggests that β2GPI might be involved in the regulation of cholesterol metabolism in a sex-specific manner. We next examined the ApoE to ApoAI ratio in the HDL fractions. Apo AI is the primary protein component of HDL, [8] whilst 70% of Apo E is in HDL 2 and 30% in HDL 3 [26]. A detailed discussion of the biology of the different HDL subfractions is beyond the scope of this article, but briefly HDL 2 particles are larger and less dense than HDL 3 and both have been suggested to have different functions [27]. Notably, we found that the ApoE/ApoAI ratio was increased in female β2GPI KO mice compared to WT, regardless of diet, with the highest ratio observed in the HF diet group. No difference in the ratio was observed in male mice fed either diet, although the ratio was higher in both WT and KO males fed a HF diet compared to those on NC. In the female β2GPI KO HF diet group, the Apo E to Apo AI ratio resembled that of male mice on a HF diet.

We observed β2GPI within the HDL fractions of female WT mice and its density increased when the mice were fed a HF diet. Additionally, β2GPI directly interacts with Apo E, as demonstrated in the co-immunoprecipitation experiments using murine plasma. These findings suggest that in female mice, β2GPI may modulate trafficking and biological functions of Apo E in HDL.

To evaluate the relevance of our murine findings to humans, we analyzed plasma from individuals with β2GPI deficiency. We identified three completely β2GPI-deficient individuals, one female (age 88), and two males (ages 55, 85) from a larger study on coronary artery disease [17]. Complete β2GPI deficiency is rare, a study of 1150 participants found no β2GPI-deficient individuals [28]. While our small sample size limits definitive conclusions, the data from these rare individuals provides valuable insights for hypothesis generation. Notably, both female murine and human β2GPI deficient sample(s) showed increased HDL cholesterol levels and a shift to larger particles compared to controls.

This suggests that β2GPI may regulate the size of HDL particles, potentially by affecting the packing of Apo E. With the deletion of β2GPI, Apo E packing may change, leading to larger HDL particles and a shift from HDL 3 to HDL 2. A recent proteomic study of small, medium, and large HDL subclasses in 6 healthy humans and 3 rats noted that there was a reciprocal relationship between β2GPI and Apo E [14]. Beta-2 glycoprotein I ratios are highest in the small HDL fractions, and ApoE was in the lower ratio range. Whereas this was reversed in the medium and larger HDL fractions (see Fig. 4B within the manuscript of reference 6) [6]. This provides strong supporting evidence that β2GPI physiologically may be responsible for regulating HDL size in rodents and humans. 

Like in the murine experiments, Apo E co-immunoprecipitated with β2GPI when human plasma from normal control was used. Direct binding experiments using human Apo E2 determined that β2GPI binds via its 5th domain (Domain V). Domain V of β2GPI has been implicated in its pleotropic biological functions [2]. In mice there is only one form of Apo E, whereas in humans there are 3 isoforms that differ in the amino acid residues at positions 112 and 158 (Apo E2 cysteine (Cys) at both, Apo E3 (Cys) at 112 and Arginine (Arg) 158, Apo E4 Arg at both. Our findings imply that the β2GPI and ApoE interaction are ApoE isoform independent.

We hypothesize that the higher HDL cholesterol levels and the shift to an increased size observed in β2GPI KO mice may be due to impaired HDL clearance, possibly resulting from the loss of β2GPI-mediated HDL uptake or catabolism by the liver and/or kidney. The liver plays a key role in HDL uptake via several receptors on hepatocytes, including Scavenger Receptor Class B1 (SR-B1) and Low Density Lipoprotein Receptors (LDLR)) which bind larger HDL particles [29]. We observed a shift to larger HDL particles in both mouse and human β2GPI deficiency supporting an accumulation of larger particles due to decreased clearance. SR-B1 mediates the selective uptake of HDL cholesterol without degrading apolipoproteins. Apo E-rich HDL interacts with LDLR and other Apo E receptors, contributing to HDL uptake by the liver. However, even HDL lacking Apo E can be taken up and degraded by hepatocytes, suggesting alternative mechanisms for HDL clearance [30]. B2GPI, potentially in synergy with Apo E, may enhance HDL uptake by hepatocytes by interacting with one of the LDL receptors. The most prominent with which β2GPI has been shown to bind is low-density lipoprotein receptor-related protein 8 (LRP8), also known as Apolipoprotein E receptor 2 (Apo ER2) [31]. LRP 8 is enriched in the brain, particularly in the neocortex, cerebellum and hippocampus [32]. Outside the nervous system, LRP8 can be found in reproductive organs, platelets, immune cells and vascular endothelium [32]. LRP 8 inactivation has no impact on plasma triglyceride or cholesterol levels [32], hence is unlikely to be directly responsible for the altered HDL levels observed in β2GPI KO mice in the current study. Alternatively, β2GPI may also mediate HDL uptake through its interaction with heparan sulfate, [33] which promotes selective uptake of HDL 3 by hepatic lipase (HL) [34]. Furthermore, β2GPI has been shown to bind to megalin (LRP2), a receptor involved in HDL homeostasis [35, 36]. Megalin, expressed in the renal tubules, plays a role in HDL catabolism, [36] suggesting another potential pathway through which β2GPI may influence HDL regulation in our study.

The sexual dimorphism demonstrated by our data requires further investigation to discern the mechanism(s), as it is likely to be complex. A murine study assessed the distinct roles of gonadal hormones versus sex chromosomes on HDL cholesterol plasma levels using the Four Core Genotypes mouse model (XX females, XX males, XY females, and XY males) and found that there was a complex interplay influenced by both, and also high fat diet [25]. This suggests that β2GPI’s female specific role in Apo E/HDL regulation may be under multiple influences including sex chromosomes, sex hormones and diet. Future studies using hormonal manipulation models will be important to establish the relative contributions of β2GPI and sex hormones to female-specific lipoprotein remodelling. We envision future studies utilizing the Four Core Genotype mouse model in conjunction with our β2GPI KO mice. These studies are complex with multiple interactions requiring large numbers of mice to identify the relevant interactions and contributions of sex hormones versus sex chromosomes versus diet.

Previous studies have shown that Apo E promotes adiposity by inhibiting the conversion of white adipose tissue to thermogenic beige adipose tissue [37–39]. In contrast, thermogenic stimulation of brown and beige adipocytes has been found to promote HDL cholesterol clearance [40]. In previous studies, we observed that female C57BL/6 β2GPI KO mice, when fed a high-fat diet, lost their resistance to developing visceral adiposity compared to WT female mice [42]. Notably, the β2GPI KO female mice exhibited a male-like obesity phenotype and weight gain profile, whereas no differences were observed in male WT versus male β2GPI KO mice [41]. This finding is clinically relevant, as increased visceral adiposity is associated with a higher risk of early mortality [42]. A genetic study found that a polymorphism in the Fat Mass and Obesity-associated (FTO) gene, in conjunction with a β2GPI polymorphism, conferred a protective effect against obesity [43]. The FTO region is strongly associated with obesity and the modulation of adipocyte thermogenesis [44]. Integrating all the above, including our current findings, we speculate that β2GPI may promote beige fat development in females by inhibiting Apo E’s effects on adipocytes. This may explain the resistance to weight gain and higher HDL turnover in female WT mice. In the absence of β2GPI, Apo E function is restored, leading to increased visceral fat and decreased HDL turnover, similar to the male phenotype. This mechanism could have evolutionary significance, given the different pressures on females and males.

Future studies should investigate the interaction between β2GPI and Apo E isoforms (Apo E2, Apo E3, and Apo E4), given their roles in neuroinflammation and disease. For instance, we have shown that female β2GPI KO mice, especially when fed a high-fat diet, exhibit increased activation of the MyD88 pathway in the hypothalamus, which plays a critical role in neuroinflammation during obesity and aging [41, 45]. This pathway is activated downstream of Toll like receptor 4 (TLR4), a key receptor for lipopolysaccharide (LPS). Apo E isoforms modulate neuroinflammation in different ways: Apo E4 knock-in mice, for example, show increased glial activation and cytokine production after LPS exposure, along with a loss of synaptic proteins, compared to Apo E3 and Apo E2 knock-in mice [46]. We hypothesize that β2GPI may play a key role in linking visceral fat accumulation to Alzheimer’s risk, [47] which warrants further investigation. Previous clinical studies have revealed sex- and age-related differences in the association of apolipoproteins with cognitive performance. Notably, only women exhibited significant negative correlations between Apo B, Apo E, β2GPI (Apo H), and Apo J levels and cognitive performance in mid-life [48]. These findings underscore the potential sex-specific role of β2GPI in influencing cognitive health, particularly in relation to Alzheimer’s disease risk. Additionally, obesity itself may further accentuate sex differences in dementia risk. A 27-year longitudinal study reported that mid-life obesity increases dementia risk significantly more in women than in men [49]. Obese women were twice as likely to develop dementia compared to women of normal weight, whereas obese men only exhibited a nonsignificant increase in risk. Similar patterns were observed with overweight status, suggesting that women may have a heightened vulnerability to the cognitive consequences of adiposity. These results align with the sexually dimorphic mechanisms we propose for β2GPI and Apo E, further emphasizing the need for sex-specific approaches in both obesity and neurodegenerative disease research.

β2GPI is a major autoantigen in the APS [50] and is a common autoantigen in systemic lupus erythematosus (SLE), both of which are autoimmune diseases associated with vascular pathology and accelerated atheroma formation [51, 52]. SLE primarily affects women of childbearing age, leading to early morbidity and mortality due to vascular complications [53, 54]. Given our findings that β2GPI regulates Apo E within HDL, these autoimmune conditions may provide a context for understanding how β2GPI’s role in HDL function could influence disease outcomes, particularly in females. Our findings have important implications for autoimmune diseases like APS and SLE, as we have shown that β2GPI is a key component of HDL cholesterol and regulates Apo E levels within HDL in females. This may explain why autoantibodies to β2GPI are often found to correlate with autoantibodies to HDL in patients with APS and SLE [54]. We speculate that anti-β2GPI autoantibodies may target β2GPI on the surface of HDL, potentially impairing its antioxidant function. Additionally, studies with Apo E knockout mice infused with anti-β2GPI autoantibodies have demonstrated accelerated atheroma formation [55]. We hypothesize that the absence of Apo E in HDL may result in higher β2GPI levels on the HDL surface, increasing autoantibody binding and impairing HDL function, which could contribute to accelerated atheroma formation in these diseases. The Apo E deficient mouse model highlights significant sex-specific differences in autoimmune pathology. Female Apo E deficient mice showed more severe autoimmune features than males, including higher ANA and anti-dsDNA autoantibodies, enlarged spleens, and increased immune complex depositions (IgG and C3) in kidneys and aortic plaques [56]. These findings mirror the female predominance in human SLE and suggest that sex hormones amplify immune activation.

A high-fat diet further exacerbated autoimmune manifestations in Apo E deficient mice, underscoring the interplay between diet, autoimmunity, and vascular pathology. These insights emphasize the need for sex-specific research to understand the interactions of β2GPI, Apo E, and autoantibodies in autoimmune disease and vascular outcomes.

A limitation of this study is that the sample size (*n* = 5 mice per sex/genotype/diet group) is not sufficient to robustly power a three-way ANOVA. While such a factorial approach would be ideal for formally testing main effects and interactions of sex, genotype, and diet, our current dataset is only powered to detect large interaction effects. For this reason, we prespecified sex-stratified analyses and focused on within-sex genotype contrasts, which directly address the biological question of female-specific β2GPI effects. Future studies with larger cohorts (*n* ≈ 10–12 per group, total *N* ≈ 80–96) will be necessary to fully resolve higher-order interactions.

### Perspectives and significance

In conclusion, our findings identify β2GPI as a crucial new regulator of Apo E containing HDL cholesterol, particularly in females. These insights have significant implications for our understanding of Apo E/HDL related biology in both health and disease, especially in the context of autoimmune associated cardiovascular disease and neurodegenerative disease like Alzheimer’s, which predominantly affect females.

## Data Availability

No datasets were generated or analysed during the current study.
